# Internet Skills Performance Tests: Are People Ready for eHealth?

**DOI:** 10.2196/jmir.1581

**Published:** 2011-04-29

**Authors:** Alexander J A M van Deursen, Jan A G M van Dijk

**Affiliations:** ^1^University of TwenteDepartment of Media, Communication and OrganizationEnschedeNetherlands

**Keywords:** Internet, eHealth, online, skills, literacy, information, digital divide

## Abstract

**Background:**

Despite the amount of online health information, there are several barriers that limit the Internet’s adoption as a source of health information. One of these barriers is highlighted in conceptualizations of the digital divide which include the differential possession of Internet skills, or “eHealth literacy”. Most measures of Internet skills among populations at large use self-assessments. The research discussed here applies a multifaceted definition of Internet skills and uses actual performance tests.

**Objective:**

The purpose of this study was to assess how ready a sample of the general population is for eHealth. More specifically, four types of Internet skills were measured in a performance test in which subjects had to complete health-related assignments on the Internet.

**Methods:**

From November 1, 2009, through February 28, 2010, 88 subjects participated in the study. Subjects were randomly selected from a telephone directory. A selective quota sample was used divided over equal subsamples of gender, age, and education. Each subject had to accomplish assignments on the Internet. The Internet skills accounted for were categorized as operational (basic skills to use the Internet), formal (navigation and orientation), information (finding information), and strategic (using the information for personal benefits). The tests took approximately 1.5 hours and were conducted in a University office, making the setting equally new for all. Successful completion and time spent on the assignments—the two main outcomes—were directly measured by the test leader.

**Results:**

The subjects successfully completed an average of 73% (5.8/8) of the operational Internet skill tasks and an average of 73% (2.9/4) of the formal Internet skill tasks. Of the information Internet skills tasks, an average of 50% (1.5/3) was completed successfully and, of the strategic Internet skills tasks, 35% (0.7/2). Only 28% (25/88) of the subjects were able to successfully complete all operational skills tasks, 39% (34/88) all formal skills tasks, 13% (11/88) all information skills tasks, and 20% (18/88) both the strategic skill tasks. The time spent on the assignments varied substantially. Age and education were the most important contributors to the operational and formal Internet skills. Regarding the formal Internet skills, years of Internet experience also had some influence. Educational level of attainment was the most important contributor to the information and strategic Internet skills.

**Conclusions:**

Although the amount of online health-related information and services is consistently growing, it appears that the general population lacks the skills to keep up. Most problematic appear to be the lack of information and strategic Internet skills, which, in the context of health, are very important. The lack of these skills is also problematic for members of younger generations, who are often considered skilled Internet users. This primarily seems to account for the operational and formal Internet skills. The results of the study strongly call for policies to increase the level of Internet skills.

## Introduction

The Internet nowadays functions as an important source of health information for the general population. The use of websites in the domain of health care varies from searching for information, getting support from Internet-based peer groups, getting online consultations, and obtaining health interventions [1]. The most commonly reported function is searching for health information [2]. People often search for health information on the Internet when prescribed a new medication or course of treatment, when dealing with a medical condition, when having unanswered questions after a doctor’s visit, or when deciding to change diets or exercise habits [3]. Popular topics include fitness, drugs, hospitals, treatments, alternative medicines, and doctors [4-6]. Among the potential benefits of providing health information online are saving time and effort, easier access, getting help when feeling embarrassed or stigmatized, healthier lifestyles, early detection of potential medical problems, collaborative treatment of illnesses, and access to treatments that a local provider may not have access to [1,7].

To realize the Internet’s potential for improving the health of the public, there are some important drawbacks that should be accounted for: (1) there are only a few tools available to help people find relevant information among the excessive amount of information that is available [8]; (2) the available information is often incomplete [8]; (3) the scientific quality of online health information is often questionable, and there are several examples of the supply of potentially harmful information on topics such as cancer rates, smoking cessation methods, and fever management in children [9-11]; and (4) users must be able to understand the information found and put it into practice [12]. Difficulties with technical terms and required reading skill levels appear to be problematic [8]. All these barriers that limit the Internet’s widespread adoption as a source of health information relate to one of the most important factors in the current digital divide debate. While early research on the digital divide primarily focused on a binary classification of having or not having physical access to the Internet, a more refined understanding has appeared with the differential possession of Internet skills as key aspect [13-16].

### Levels of Internet Skills 

Deficient levels of Internet skills may prevent people from recognizing that information is missing, from understanding the difference between biased and unbiased information, from distinguishing evidence-based claims, and from interpreting the information intended for health professionals [17]. An indication of the low levels of search-related Internet skills is that one of the most common complaints about online health information searches is the amount of time required to process the documents that are found online [18]. While Internet search engines help to identify a large number of health-related documents, use of search engines calls for advanced skills that not all information consumers may possess [19]. In general, search engines and simple search terms do not seem to provide efficient access to health information [20]. Furthermore, most users seem to focus on finding information quickly rather than on evaluating the information found [21]. Most people only explore the first few links obtained from a search using a general search engine [22].

Besides search-related problems, more basic problems also limit the general public’s use of online health information and services. Actual performance tests in the United States revealed that the general user population in the United States lacks an understanding of the basics of surfing the Internet [23]. In the Netherlands, the results of performance tests that have been conducted support the search-related problems described above [24]. Furthermore, these tests also revealed that a large part of the population struggles with deficiencies of other Internet skills [24]. Not everyone seems to be able to save or even open PDF documents. In addition, the basic operations needed to access the Internet such as typing a URL in the address bar or typing search queries in the search bar are not self-evident for all Internet users. Furthermore, many Internet users experience problems with website designs and when new browser windows are opened, something not unusual on the Internet.

### Measuring Internet Skills

The literature concerning Internet skills is not consistent in the terms used or in the underlying concepts applied. Though there are many converging views, there is no agreement on the exact definition. The types of Internet skills that have received the most of the attention are basic technological knowledge and search behavior. In health-related literature, a popular concept is health information–seeking behavior [25]. Unfortunately, most studies provide little insight into this concept’s specific meaning [25]. Contrary to the many related definitions of Internet skills, only a few measurable dimensions seem to have been applied within specific settings. One exception can be found in the field of health care. Here, the 8-item eHealth Literacy Scale (eHEALS) was developed to measure consumers’ combined knowledge, comfort, and perceived skills at finding, evaluating, and applying electronic health information to health problems [26]. The scale was based on a model that distinguishes between six types of literacy that are organized into analytic (traditional, media, information) and context-specific (computer, scientific, health) types. Although several skills required for the general Internet user population are considered, the main problems with this instrument are that the scale lacks validation and is based on an individual’s perception of one’s own skills or knowledge. This method is used in almost all strategies for measuring Internet skills for the obvious reason that observational studies are time-consuming and expensive. Unfortunately, measurements that use surveys and self-reports have significant problems of validity [27,28].

There are few measurements and scientific investigations of the actual levels of Internet skills possessed by populations at large [23,24,28]. We draw upon the definitions of Internet skills developed by Van Deursen and Van Dijk to encourage researchers to focus on in-depth skills measurements [24,28]. Van Deursen and Van Dijk elaborated and validated four types of Internet skills from an extensive literature overview. These are termed operational, formal, information, and strategic Internet skills.

Operational Internet skills indicate a set of basic skills in using an Internet browser, search engine, or Web-based form.

Formal Internet skills relate to the hypermedia structure, which requires the following skills: (1) navigating through different Web and menu layouts and (2) keeping a sense of orientation (disorientation is the most frequently cited problem in hypermedia use [29]).

Information Internet skills are derived from staged approaches in explaining the actions via which users try to fulfill their information needs [30]. Some of these actions are present in studies concerning health information–seeking behavior. However, in these studies, it is often difficult to locate explicit definitions. The actions considered here are: problem definition, choosing a way of searching, defining search queries, selecting relevant information, and evaluating the information found.

Strategic Internet skills enhance the capacity to use the Internet as a means of reaching particular goals including the general goal of improving one’s position in society. Strategic Internet skills are derived from the classical approach to decision making, where emphasis lies on procedures through which decision makers can reach optimal solutions as efficiently as possible [31].

By classifying the four Internet skills into medium-related Internet skills (operational and formal) and content-related Internet skills (information and strategic), Van Deursen and Van Dijk avoid a technological deterministic viewpoint since the command of hardware and software is not the only focus of attention. Both technical aspects related to the use of the Internet and substantial aspects related to the content provided by the Internet are accounted for. Furthermore, the four Internet skills have a sequential and conditional nature [28]. They illustrate that the possession of operational and formal skills alone is a necessary but not sufficient condition when using the Internet. Table 1 shows the specific skills included in each of the four Internet skill categories.

**Table 1:** Internet skills definitions [24,28]

Skill Category		Specific Skills
**Medium-related Internet skills**		
	Operational skills in using Internet browsers	Opening websites by entering the URL in the location bar Navigating forward and backward between pages using the browser buttons Saving files on the hard disk Opening various common file formats (eg, PDFs) Bookmarking websites Changing the browser’s preferences Using text or images with hyperlinks
	Operational skills using Internet-based search engines	Entering keywords in the proper field Executing the search operation Opening search results in the search result lists
	Operational skills operating Internet-based forms	Using the different types of fields and buttons Submitting a form
	Formal skill used to navigate on the Internet	Using hyperlinks embedded in different formats such as texts, images, or menus
	Formal skill in maintaining a sense of location while navigating	Not becoming disoriented when navigating within a website Not becoming disoriented when navigating between websites Not becoming disoriented when opening and browsing through search results
**Content-related Internet skills**		
	Information skills used to locate required information	Choosing a website or a search system to seek information Defining search options or queries Selecting information on websites or in search results Evaluating information sources
	Strategic skills used to take advantage of the Internet	Developing an orientation toward a particular goal Taking the right action to reach this goal Making the right decision to reach this goal Gaining the benefits resulting from this goal

The purpose of this study was to assess how ready the general population is for the transformation of information and services to the Internet in the domain of health care. The Internet skills of the Dutch population were measured in a performance test in which subjects had to complete health-related assignments on the Internet. The first research question was: What are the levels of Internet skills of Dutch citizens when using the Internet in the domain of health care? 

When measuring Internet skills, the following variables should be accounted for [32]: Gender, age, educational attainment, time spent online, years of Internet experience, social resources, socioeconomic position, the location of Internet use, and participation in an Internet course. The second research question was: What are the characteristics of individuals who are most likely to suffer from inadequate levels of Internet skills in the domain of health care?

## Methods

### Recruitment

Subjects were recruited using random digit dialing in cities and villages in the region of Twente in the eastern region of the Netherlands. This region is fairly representative of the country as a whole because the demographics of the population as well as the proportion of the population living in rural versus urban settings are similar to those in the country as a whole. In line with procedures applied in prior research [24,28,32], a condition of participation was use of the Internet at least once every month for purposes other than email alone. Although this condition excluded approximately 20% of the Dutch population, it ensured that low frequency users who are nevertheless familiar with the Internet were also included. The invitation policy put people who feared a test at ease. Only Dutch-speaking adults 18 years of age and older were included and were promised €25 for their participation in a one-and-a-half hour research session.

To increase the representativeness of the findings, the subjects were recruited by applying a stratified random sampling method. First, a sample was randomly selected from a telephone directory. Subsequently, a selective quota sample was drawn from the strata to reach equal subsamples of gender, age (equal number of subjects in the categories of age 18-29, 30-39, 40-54, and 55-80), and educational level of attainment (equal number of subjects in the categories low, middle, and high). The result of this sampling procedure is that the results are not representative of the whole Dutch population. The focus is on the relative differences between the subsamples in terms of relative skill levels by age, gender, and education controlling for variables such as Internet experience. When respondents indicated they were willing to participate, their contact and email address were recorded and a time for the research session was scheduled. Respondents received a follow-up letter in the mail for confirmation and with directions to the research site. The day before the study, respondents were reminded of the session by phone. 

### Procedure

The performance tests were conducted from November 1, 2009, through February 28, 2010, in a university office. Prior to the test, a 10-minute questionnaire was administered to gather personal data. Subjects were asked for their year of birth, gender, educational level of attainment, amount of Internet use (hours per week), Internet experience (in years), location of respondents' regular Internet use, social support networks, and socioeconomic status.

In the performance tests, subjects used a keyboard, a mouse, and a 17-inch monitor connected to a laptop with a high-speed Internet connection. The laptop was programmed with the three most popular Internet browsers (Internet Explorer, Mozilla Firefox, and Google Chrome), which allowed the subjects to replicate their regular Internet use. No default page was set on the browsers, and all the assignments started with a blank page. To ensure that subjects were not influenced by a previous user’s actions, the browser was reset after each session by removing temporary files, cookies, and favorites. In addition, downloaded files, history, forms, and passwords were removed and the laptop was rebooted.

During the assignment completion, subjects themselves decided when they were finished or wanted to give up on an assignment. No encouragements were given because the pressure to succeed was already higher in the laboratory setting than at home. After a specified ample amount of time had passed (determined for every task based on the results of 12 pilot tests), the test leader gently asked the subjects to move on to the next assignment (see Multimedia Appendix 1 for a complete overview of the assignments, the corresponding Internet skills, and the maximum time allowed). If the correct answer was not found, the assignment was rated as not completed. Both successful completion and time required—the main outcomes of the performance test—were directly noted during the sessions.

### Assignments

The assignments the subjects had to complete were all health-related and accessible to the general user population. All assignments were fact-based and had a specific correct action or answer. Open-ended tasks were avoided because of the ambiguity of interpretation of the many potential answers. Included were two assignments (consisting of 8 tasks) to measure operational Internet skills, two (consisting of 4 tasks) to measure formal Internet skills, three to measure information Internet skills, and two to measure strategic Internet skills. In the operational assignments, subjects were, for example, asked to open a health website, save a file, or add a website to the “favorites.” Examples of tasks in the formal skills assignments were navigating different health-related menu and website designs and surfing between different websites. The information skill assignments charged subjects with finding health-related information on the Internet (requiring the skills described in Table 1 to locate information). Subjects were, for example, asked to find the name of a specific medical condition or to find out whether it would be a good idea to start with a treatment after being infected. Finally, the strategic skill assignments forced subjects to extract information from different sources, make decisions based on the information found, and gain personal benefits by making the right decisions. For example, subjects were asked to find out whether it would be a good idea to give a 3-year-old boy Vitamin A and D supplements, or to find a homecare organization with a specific caring program. All assignments were pilot tested with 12 subjects to ensure comprehensibility and applicability.

## Results

### Characteristics of the Sample Population

The characteristics of the subjects that participated in the performance test are shown in Table 2. The average number of years of Internet experience reported was 9.3 (SD 4.3) and the average amount of Internet use reported was 12.2 (SD 13.7) hours per week. Overall, the people who participated represented a diverse group of Internet users.

**Table 2:** Distribution of subjects by demographic variables (gender, education, and age) and the control variables (location of Internet use, needing assistance, socioeconomic status, and participation in an Internet course)

Characteristic		n (%)
**Gender**		
	Male	45 (51%)
	Female	43 (49%)
**Age**		
	18-29	24 (27%)
	30-39	18 (21%)
	0-54	23 (26%)
	55-80	23 (26%)
**Level of education (highest completed)**		
	Low (primary school)	25 (28%)
	Middle (high school)	32 (36%)
	High (college or university)	31 (35%)
**Primary location of Internet use**		
	At home	75 (85%)
	At work	1 (1%)
	At school	8 (9%)
	At friends or family	3 (3%)
	At a library	1 (1%)
**Assistance when using the Internet**		
	No	49 (56%)
	Yes, from family	18 (21%)
	Yes, from friends	17 (20%)
	Yes, from colleagues	4 (5%)
	Yes, from a helpdesk	0 (0%)
**Socioeconomic status**		
	Employee	30 (34%)
	Retired	14 (16%)
	Student	21 (24%)
	Housemen/housewife	4 (4%)
	Employer	6 (7%)
	Disabled	4 (5%)
	Unemployed	9 (10%)
**Participation in an Internet course**		
	No	63 (72%)
	Yes	25 (28%)

### Task Completion and Time Spent

Table 3 shows that the subjects successfully completed an average of 5.8 (73%) of the 8 operational Internet skill tasks and an average of 2.9 (73%) of the 4 formal Internet skill tasks. Of the 3 information Internet skills tasks, an average of 1.5 (50%) were completed successfully, and of the 2 strategic Internet skills tasks, an average of 0.7 (35%) was completed successfully. The time spent on all assignments varies substantially.

**Table 3:** Overview of successful task completion and time spent

Internet Skills (Number of Tasks)	Average Task Completion		Seconds Spent	
	Mean (SD)	%	Mean (SD)	Minimum/Maximum time
Operational tasks (8)	5.8 (2.1)	73	427 (198)	118/980
Formal tasks (4)	2.9 (1.2)	73	450 (218)	180/1143
Information tasks (3)	1.5 (0.9)	50	960 (336)	343/1717
Strategic tasks (2)	0.7 (0.8)	35	1613 (545)	441/2500

**Table 4:** Number of tasks subjects failed to complete successfully

	Number of Failed Tasks	Number of Subjects (%)
Operational Internet skills	0	25 (28%)
	1	18 (21%)
	2	14 (16%)
	3	9 (10%)
	4	9 (10%)
	5	8 (9%)
	6	4 (4%)
	7	3 (3%)
	8	0 (0%)
Formal Internet skills	0	34 (39%)
	1	25 (28%)
	2	15 (17%)
	3	10 (11%)
	4	4 (5%)
Information Internet skills	0	11 (13%)
	1	35 (40%)
	2	30 (34%)
	3	12 (14%)
Strategic Internet skills	0	18 (20%)
	1	31 (35%)
	2	39 (44%)

Table 4 reveals that 28% of the subjects (25/88) were able to complete all operational Internet skills tasks, 39% (34/88) all formal Internet skills tasks, 13% (11/88), all information Internet skills tasks, and 20% (18/88), both of the strategic skills tasks. Furthermore, 44% of the subjects (39/88) could not complete either of the 2 strategic Internet skills tasks successfully. This was 13% (11/88 subjects) regarding the 3 information Internet skills tasks. The second strategic Internet skills tasks was the hardest and could only be completed successfully by 25% of the subjects (22/88). In this task, subjects were asked to find a homecare organization in the city of Enschede with a special caring program for individuals suffering from dementia and impaired hearing.

### Contributors to the Level of Internet Skills

To identify factors that contribute to the level of Internet skills, two linear regressions for all 4 skills were conducted: one with the number of assignments completed and one with the time spent on these assignments as dependent variable. The independent variables in the regression model were gender, educational level attained (coded from 1, low to 3, high), age (years since birth), Internet experience (years online), amount of time spent on the Internet (hours per week), using social support (yes vs no), the primary location of Internet use (at home vs elsewhere), and socioeconomic status (active vs inactive). 

Table 5 contains the linear regression results of the number of operational tasks completed successfully (*R* ^2^ = .55, *F*_9,87_ = 10.11, *P <* .001) and the time spent (*R* ^2^ = .54, *F*_9,87_ = 10.11 (*P <* .001). Age and education are the two significant contributors to the number of operational Internet skills tasks successfully completed and to the time spent on these tasks. Age is the strongest contributor. For seniors and people with lower levels of education, the most problematic task was saving PDF files. 

**Table 5:** Linear regression results of the number of operational tasks completed successfully and the time spent

Independent Variables	Number of Tasks Completed		Time Spent	
	Beta	*P*	Beta	*P*
Gender (male/female)	−.10	.21	−.02	.65
Age (in years)	−71	< .001	.51	< .001
Education (low to high)	.18	.04	−.27	.01
Internet experience (in years)	.12	.17	−.15	.09
Time online (hours per week)	.02	.85	−.04	.64
Followed an Internet course (no/yes)	.02	.83	.10	.33
Using peers for help (no/yes)	.03	.72	.08	.32
Primary location of use (at home/elsewhere)	.01	.87	−.09	.28
Working situation (inactive/active)	−.11	.27	−.06	.59

Table 6 contains the linear regression results of the number of formal tasks completed successfully (*R* ^2^ = .61, *F*_9,87_ = 13.60, *P <* .001) and the time spent (*R* ^2^ = .68, *F*_9,87_ = 18.20, *P <* .001). Again age and education were significant contributors to both the number of successfully completed tasks and the time spent. In addition, number of years of Internet experience contributed significantly to both equations. Again seniors and people with lower levels of education experienced the most problems, especially with navigating different Web layouts.

**Table 6:** Linear regression results of the number of formal tasks completed successfully and the time spent

Independent Variables	Number of Tasks completed		Time Spent	
	Beta	*P*	Beta	*P*
Gender (male/female)	−.09	.27	.00	.36
Age (in years)	−.62	< .001	.61	< .001
Education (low to high)	.40	< .001	−.34	< .001
Internet experience (in years)	.17	.04	−.15	.03
Time online (hours per week)	−.11	.16	.00	.33
Followed an Internet course (no/yes)	−.05	.63	.07	.37
Using peers for help (no/yes)	−.06	.38	.03	.28
Primary location of use (at home/elsewhere)	.07	.32	−.04	.22
Working situation (inactive/active)	−.13	.18	−.02	.19

Table 7 contains the linear regression results of the number of information tasks completed successfully (*R* ^2^ = .34, *F*_9,87_ = 4.48, *P <* .001) and the time spent (*R* ^2^ = .09, *F*_9,87_ = 1.71, *P <* .001). Educational level of attainment is the strongest significant contributor to the number of information tasks completed successfully. Furthermore, participation in an Internet course positively contributes to the number of tasks completed successfully, and using peers for help negatively contributes significantly to the time spent on the tasks. The most difficult information Internet skills task was completed by only 28% (25/88) of the subjects. This task asked the subjects whether it is a good idea to start an antiviral (remedy against viral infections) for Lyme borreliosis. Subjects with lower levels of education, in particular, used broad search queries (eg, searching for the word *tick*) and did not venture past the first 3 search results. Also remarkably was that almost none of the subjects evaluated the information found. 

**Table 7:** Linear regression results of the number of information tasks completed successfully and the time spent

Independent variables	Number of asks Completed		Time Spent	
	Beta	*P*	Beta	*P*
Gender (male/female)	.09	.26	−.13	.19
Age (in years)	−.06	.72	.08	.37
Education (low to high)	.56	< .001	−.08	.63
Internet experience (in years)	.01	.50	−.08	.50
Time online (hours per week)	−.01	.91	.00	.98
Followed an Internet course (no/yes)	−.24	.04	−.00	.77
Using peers for help (no/yes)	−.04	.46	.28	.01
Primary location of use (at home/elsewhere)	−.09	.50	−.05	.60
Working situation (inactive/active)	−.00	.71	−.04	.76

Table 8 contains the linear regression results of the number of strategic tasks completed successfully (*R* ^2^ = .45, *F*_9,87_ = 7.10, *P <* .001) and the time spent (*R* ^2^ = .07, *F*_9,87_ = 0.62, *P* *=* .21). Educational level of attainment is the only significant contributor to the number of successfully completed strategic Internet skills tasks. Hours spent online weekly and Using peers for help appeared to be significant contributors to the time spent on the tasks. The second strategic Internet skills tasks was the most difficult and could only be completed successfully by 25% of the most highly educated subjects (22/88). In this task, subjects were asked to find a homecare organization in the city of Enschede with a special caring program for people with dementia and impaired hearing.

**Table 8:** Linear regression results of the number of strategic tasks completed successfully and the time spent

Independent Variables	Number of Tasks Completed		Time Spent	
	Beta	*P*	Beta	*P*
Gender (male/female)	.11	.11	.11	.11
Age (in years)	.01	.55	.17	.14
Education (low to high)	.58	< .001	.11	.12
Internet experience (in years)	.07	.52	−.14	.15
Time online (hours per week)	.03	.63	.02	.54
Followed an Internet course (no/yes)	−.05	.89	−.06	.44
Using peers for help (no/yes)	−.00	.74	−.03	.46
Primary location of use (at home/elsewhere)	.02	.70	−.13	.22
Working situation (inactive/active)	.12	.22	.10	.20

## Discussion

### Principal Results

This study examined the level of Internet skills of a sample of the Dutch population when using the Internet for health-related information and services. Furthermore, it was examined whether skill levels can be predicted by demographic and socioeconomic factors. The study applied an in-depth definition of Internet skills by distinguishing between operational, formal, information, and strategic Internet skills. All four types of skills were measured in an actual performance test. While the test is not statistically representative for the general Dutch population, the results suggest that the sample on average possesses a sufficient level of operational and formal Internet skills when using the Internet for health-related topics. However, the levels of information skills and especially strategic Internet skills attained are probably much lower. Age and educational attainment are the most important contributing factors. Age appeared significant for the levels of operational and formal Internet skills, but not for the levels of information and strategic Internet skills.

Especially in the domain of health care, having sufficient levels of information and strategic skills is very important since the quality of the information offered is often questionable and unfortunately too often seems to be taken for granted. This might even mean that the lack of information and strategic Internet skills can become vital in the most literal sense. This happens when people with lower levels of Internet skills cannot find the hospital with the shortest waiting list for surgery or the best qualifications. This also occurs when they lack any other crucial information that helps them in preventing or relieving a particular urgent disease or when they are not able to ask for a second opinion about a proposed treatment. This is alarming, especially when considering that outside the artificial test situation created here, performance might be even lower (although we did not explicitly encourage the subjects). 

It appears that the younger generations also are in need of improved information and strategic Internet skills. Younger generations are often considered to be skilled users of the Internet. In contrast to this, older people are often regarded as lagging behind in the adoption of new innovations. Together with the difficulties in learning new skills, resistance to change has also been suggested as a barrier to Internet use by the elderly [33]. However, in the performance tests reported here, it appears that younger generations do not score better on the information and strategic Internet skills. Similar results appeared in performance tests conducted in settings outside the domain of health [28,32]. This is an important finding, since there are also innumerable health sources on the Internet relevant for the younger generations. The results of the performance tests raise doubts as to whether younger people have sufficient information and strategic Internet skills to benefit from online health information provision. The same accounts for people with lower levels of education. Educational attainment appears very important in predicting who is likely to command low levels of Internet skills. Educational level of attainment proved significant for operational, formal, information, and strategic skills. Often, education is considered to be the most consistent global predictor for the use of information and communication technologies. More highly educated people are more likely to own computers, have Internet access at home, connect through broadband, and spend more time online [34]. Furthermore, more highly educated people are able to keep up with technological advancements and therefore increase their lead over people who are not able to keep up [35].

People who spend more time online—whether at work or any other location—are expected to acquire more knowledge about the Internet and are thus expected to have better online skills [27]. Moreover, people who have been Internet users for a longer period of time are presumed to be better at finding information online because they have more experience to draw on [27]. However, we found that operational, information, and strategic Internet skills do not grow with years of Internet experience and amount of time spent online weekly. Internet experience only had an effect on the formal Internet skills. Of all other variables, participation in an Internet course had some minor positive influence on the level of information Internet skills, and getting help from peers a negative influence on the same skills.

### Limitations

This study provides an overview of the levels of four types of Internet skills among different segments of the Dutch population. However, only the absolute levels of the four types of Internet skills are considered. The specific aspects of the subjects’ skills—for example the different steps concerning information or strategic Internet skills—are not considered here. A future qualitative analysis is required to provide more details about the specific skills indices.

A second limitation is that in this study, Internet use was limited to information retrieval. Communication skills were not measured because this would have made the performance tests that already required 1.5 hours from the subjects an unrealistic effort. Furthermore, content creation and sharing have also been ignored. These activities refer to so-called Web 2.0 applications. We consider information and strategic Internet skills as crucial for these activities, even more so than for information retrieval. Active participation and user-generated content require a high level of Internet skill, particularly for “serious” as compared to entertainment applications. Both limitations are a job for future researchers developing operational definitions and measurements of skills of communication, interaction, and peer-to-peer networking on the Internet.

Because of the major labor intensity of performance tests and the very high travel costs of drawing subjects to the university lab nationwide, it was not possible to test a random sample of 1200 people from the whole Dutch population. Ultimately, 88 subjects participated in the performance tests. Although this is not enough to generalize to the whole population, the applied quota sample for the categories of gender, age, and education greatly improved representativeness. Furthermore, to rate the overall representativeness of the sampling approach, the approach should be compared with the standards of an experiment rather than a survey. For an experiment, the number of subjects in our study was quite high. But larger than average experimental groups were required because large social and cultural differences in computer use and experience had to be taken into account.

### Conclusions

In conclusion, this study found that operational and formal Internet skills are not sufficient when using the Internet for health purposes. The information and strategic Internet skills that are very important for seeking health information and for making decisions based on the retrieved information appear to be quite problematic. People with lower levels of education in all age groups seem to be the most likely candidates for lacking these Internet skills. The results of this study strongly call for policies that try to improve the level of Internet skills. The gap between the content provided and the content that people are able to manage must be acknowledged and remedied [26]. This is possible by accounting for Internet skills from a demand (user) and from a supply (Internet developers) perspective [36].

From a demand perspective, it appears that systematic training of operational and formal Internet skills in all types of adult education and computer classes would benefit older generations. If people in older groups improve these skills, they are likely to perform better on the information and strategic Internet skills than the younger generations. Training in information and strategic Internet skills should be accounted for in educational programs and other training environments. Unfortunately, learning to use the Internet is not a standard component of the current curriculum in education. On the contrary, it is generally believed that technologies such as the Internet by themselves empower learners and are regarded as an easy fix to learning itself [37]. This, however, is dubious and research on the matter is often misrepresented [37]. 

From a supply perspective, further research should be conducted into strategies to improve health information and service provision on the Internet. Results of this research indicate that seniors will probably benefit the most when websites require low levels of operational and formal Internet skills. After all, seniors do not seem to be inferior to younger citizens on either information or strategic skills. The lack of information and strategic Internet skills point to a major need for the improvement of the provision of online health-related information and services. Unfortunately the quality of online information varies substantially and the excessive amount of information offered online only makes the relevant sources harder to find for many users.

## Multimedia Appendix 1

Assignments

## Abbreviations

eHEALS: eHealth Literacy Scale
