# Supplementary material for: Internet Skills Performance Tests: Are People Ready for eHealth?
Source: J Med Internet Res. 2011 Apr 29;13(2):e35. doi: 10.2196/jmir.1581 (PMC3221376; doi:10.2196/jmir.1581)
Supplement: Supplementary file 1 [file jmir_v13i2e35_app1.pdf]

Multimedia Appendix 1 - Assignments

Operational Internet skills

Assignment 1 (max time allowed: 12 minutes)

1. Go to the Web site of the RIVM (www.rivm.nl).
2. Click on the link ‘Infectieziekten’ in the menu on the left.
   Click on the subject ‘Thema’s’.
   Click on the subject ‘Hoofdluis’.
   Click on the subject ‘Voorlichtingsmateriaal’
3. Open the brochure ‘Veelgestelde vragen’
   Save the brochure in the ‘My Documents’ folder.
4. Use the back button to go to homepage of the RIVM Web site.
5. Add the homepage to the Favourites (or bookmarks)

Assignment 2 (max time allowed: 8 minutes)

6. Go to the Web site of MinVMS (www.minvws.nl).
   Click on the link ‘Uitgebreid zoeken.’
   Complete the fields using the information given below.
7. Execute the search operation and open the third search result.
8. Save the logo of the MinVWS on the desktop of the computer.

Formal Internet skills

Assignment 3 (max time allowed: 10 minutes)

1. Go to the website of ZonMW (www.zonmw.nl).
   Follow the options Onderwerpen / Jeugd / Zorg
   Choose the option: ‘RIVM/Jeugdgezondheid’.
2. Go to the homepage of the RIVM Web site in the new opened window.
   Go to the homepage of the RIVM website in the original window.
3. Perform a search on the ZonMW Web site with keyword ‘infectie’.
   Open the first search result.
   Open the fourth search result.

Assignment 4 (max time allowed: 10 minutes)

4. Find the addresses of the following health organizations. Use the Web sites of the organizations:
   ISALA Clinic in Zwolle (www.isala.nl).
   BOSK Organization (www.bosk.nl/).
   GGZ Enschede (www.ggznederland.nl)

Information Internet skills

Assignment 5 (max time allowed: 12 minutes)

1. Imagine… You would like to know more about H1N1 influenza. This was originally called Swine Flu. Answer the following question, using the Web site of Dokterdokter (www.dokterdokter.nl): Why is the name Swine flu not correct?

Assignment 6 (max time allowed: 12 minutes)

2. Imagine… During a hike you are bitten by a tick. A red spot appears that increases. This is a sign you have been infected with Lyme borreliosis. A friend recommends to start with an antiviral (remedy against viral infections) immediately since Lyme's disease can have very unpleasant consequences, especially when treatment starts too late! Answer the following question using a search engine: Is it a good idea to start an antiviral remedy?

Assignment 7 (max time allowed: 12 minutes)

3. Imagine… The last few months your son has been suffering problems with his back. His back shows a deviation to the left. It looks like one leg is shorter than the other, although this not appear to be the case. Answer the following question using a search engine: What is the name of the condition your son suffers from?

Strategic Internet skills

Assignment 8 (max time allowed: 12 minutes)

1. Imagine… You have a three year old son. Your mother advices you to give him vitamins A and D supplements since she believes these are required for a healthy growth. Answer the following question using the Internet: Would you give your son both extra vitamins A and D?

Assignment 9 (max time allowed: 30 minutes)

2. Imagine… Your mother is 82 years old. Lately, she has been suffering from dementia and impaired hearing. You decided to find a homecare organization in Enschede that has a special caring program for these complaints. You also would like the organization to organize daily activities. Use the Internet to find a homecare organization in Enschede that meets your demands?
